# Ethical considerations in the use of Machine Learning for research and statistics.

**DOI:** 10.23889/ijpds.v7i3.1921

**Published:** 2022-08-25

**Authors:** Alice Toms, Simon Whitworth

**Affiliations:** 1 Office for National Statistics

This paper, based upon new guidance created in collaboration with researchers from several national statistical institutes, explores the main ethical considerations associated with the use of machine learning techniques for aggregate statistics. The aim of this paper is to provide applied, practical ethical guidance for researchers using machine learning techniques.

Following an extensive literature review, alongside discussion and collaboration with a number of national statistical institutes, it was identified that there was a need for applied guidance on the use of machine learning for the production of official statistics by the international research and statistical community. Feedback was gathered from interested stakeholders, which found that whilst there were resources available to researchers relating to the ethical considerations of machine learning projects, these focus mainly on operational uses of machine learning, and furthermore, lacked advice on how to practically mitigate ethical issues that arise throughout the project lifecycle.

The guidance focuses on four main ethical considerations, found to be prevalent within machine learning research, and offers ways to mitigate these issues should they arise. These are: the importance of minimising and mitigating social bias and discrimination within machine learning research, and clearly communicating these, and the limitations of our research; the need to consider the transparency and explainability of machine learning research, and the implications this has for reproducibility; the importance of maintaining accountability throughout machine learning processes, ensuring that models are used only for their intended purposes, and that different stakeholders are aware of their responsibilities; the need to consider the confidentiality and privacy risks arising from the data used, both in relation to training data which is fed into the machine, and outputs resulting from the machine learning’s findings.

The guidance has been well-received following its release, and feedback from the wider user community to date has been positive. The IPDLN conference provides an opportunity for further feedback to be collated to ensure that the guidance continues to be valuable to its intended audience.

